# Hospital Meetings, &c.

**Published:** 1898-01-15

**Authors:** 


### The Institutional Workshop.

### HOSPITAL MEETINGS, &C.

### OPENING OF THE FRANK WRIGHT WARD AT THE LONDON TEMPERANCE HOSPITAL.

A friendly party assembled at the London Temperance Hospital on the occasion of Lady Battersea's opening this new aseptic ward.

The function took place in the charming children's ward, and the babies present were wonderfully good in spite of the unwonted invasion. Lady Battersea's little speech was bright and sympathetic. She said that lately she had heard a somewhat unkind remark to the effect that "the greatest evil drink had produced were the teetotalers." But the teetotalers had done, at any rate, one good thing in founding the Temperance Hospital. She then pronounced the new ward open, and declared that it was to be known as the "Frank Wright" ward. The usual votes of thanks were unanimously given, and the party made a tour of inspection throughout the hospital, which terminated in the board-room, where tea was served.

The new ward is a small one for one patient. The walls are opaline, with rounded corners; the floor marble mosaic; the window one huge sheet of plate glass; the stove is known as a "Teale stove," the fire lying in a well of brick and tiles without any bars. The expense of this ward is defrayed by five friends of the hospital, and the furniture is the gift of the sister of this floor. It is very handsome. The bed is a brass "Gorham," the tables and lockers are of metal and glass of the newest pattern; the utensils and toilet appurtenances are of glass and polished metal, and the chair shines like silver. The screen is metal, and hung with a full-coloured chintz. Needless to say, the ventilation is carefully considered, fresh air being forced into the room by an electric fan, and the light is electric.

The ward is named after the late Mr. Frank Wright, who joined the board in 1873, and devoted more and more of his time to the work of the hospital, and left it in his will a legacy of £500. He was vice-president at his death, and his place is now held by Mr. Vezey Strong.

Amongst those present were: Sir John Hutton, J.P., L.C.C., Mr. J. Vezey Strong, Admiral Alington, Dr. Dawson Burns, Mr. John C. Murdock, Mr. John Hilton, and the Rev. R. Faraker. Of the staff there were: J. James Ridge, M.D., B.S., B.A., and B.Sc.Lond., W. Soltan Fenwick, M.D., B.S.Lond., W. J. Collins, M.S., M.D., B.Sc., F.R.C.S., D.P.H., Mr. W. Griffith, M.B., Ch.B., and the Lady Superintendent (Miss Orme).

### NEWTON ABBOT WORKHOUSE INFIRMARY.

Lady Clifford opened the new workhouse infirmary at Newton Abbot on the 6th inst. It will accommodate 120 sick and infirm, amongst whom the imbeciles are not reckoned. It is 50 feet higher than the rest of the buildings, and stands a little in their rear. The cost of the infirmary is £10,200, and that of the laundry, workshops, bakery, and kitchens brings the expenditure up to £15,000, to which another £1,000 is added for incidental expenses.

